# Biological activity of *Dittrichia viscosa* (L.) Greuter extracts against adult *Sitophilus granarius* (L.) (Coleoptera, Curculionidae) and identification of active compounds

**DOI:** 10.1038/s41598-019-42886-4

**Published:** 2019-04-23

**Authors:** Giuseppe Rotundo, Gianluca Paventi, Antonia Barberio, Antonio De Cristofaro, Ivan Notardonato, Mario V. Russo, Giacinto S. Germinara

**Affiliations:** 1 Department of Agricultural, Environmental and Food Sciences, University of Molise, via de Sanctis, 86100 Campobasso, Italy; 2 Department of Medicine and Health Sciences “V. Tiberio”, University of Molise, via de Sanctis, 86100 Campobasso, Italy; 3 Department of the Sciences of Agriculture, Food and Environment, University of Foggia, Via Napoli 25, 71100 Foggia, Italy

**Keywords:** Entomology, Agroecology, Environmental impact

## Abstract

*Dittrichia viscosa* (L.) Greuter, a perennial weed of the Mediterranean area, was reported to be source of active substances. Here, by means of both ingestion and contact assays, the biological activity of three different extracts (n-hexane, methanol, and distilled water) of *D. viscosa* aerial part has been evaluated against *Sitophilus granarius* (L.) adults, an important pest of stored grains. Ingestion assays showed negligible mortality and food deterrence for all the extracts, whereas only a slight reduction of some nutritional parameters (relative growth rate, relative consumption rate, food efficiency conversion) was recorded for water extract. High contact toxicity was found only for the n-hexane extract (24 h median lethal dose LD_50_ = 53.20 μg/adult). This extract was further subfractioned by silica gel column chromatography and then by thin layer chromatography. Further contact toxicity bioassays highlighted two active subfractions which were analyzed by GC-MS. This revealed the occurrence, in both subfractions, of two major peaks that were identified as α- and γ- costic acid isomers. Moreover, *D. viscosa* active subfractions, did not cause acetylcholinesterase (AChE) inhibition; therefore, in the light of progressive limitation of compounds acting by this mechanism of action, *D. viscosa* represents a promising eco-sustainable source of natural products for pest control.

## Introduction

The indiscriminate use of synthetic insecticides for several decades has led to the accumulation of toxic residues in the environment and food as well as to the development of resistant pest populations^1^. A clear example of that is represented by fenitrothion, a broad-spectrum organophosphorus pesticide acting on acetylcholinesterase (AChE), which, due to its extensive use, in some regions is classified as a common river water pollutant^2^. Therefore, the search for novel eco-friendly compounds safer for customers and workers is strongly required, as also recommended by both national and international legislations. In this context, the need for effective and biodegradable pesticides has created a significant market opportunity for alternative products^3^. Botanical pesticides offer a good alternative to traditional chemicals in Integrated Pest Management (IPM)^4–6^. In fact, their use reduces the risk to non-target organisms, due to their rapid degradation in the environment, and provides novel and multiple modes of action that reduce the probability of developing pest resistance^1,7^.

In this regard, the insecticidal activity of plants belonging to the Asteraceae family has been investigated in many previous studies^8–10^. *Dittrichia viscosa* (L.) Greuter (sin. *Inula viscosa* (L.) Aiton) is a perennial Mediterranean weed species of this family reported to be a source of bioactive compounds^11,12^, as also suggested by its use in folk medicine as an antidiabetic^13^, antipyretic and anti-inflammatory treatment^14,15^. More recently, *D. viscosa* extracts were proved to have a plethora of effects going from anti HIV-activity in an *in vitro* test^16^, to an abortifacient effect^17^ or protection against *Helicobacter pylori*^18^. In particular, besides antibacterial and antifungal activities^19^, extracts from this plant showed also toxicity against the nematode *Meloidogyne javanica* (Treub) Chitiwood^20^, the mite *Tetranichus cinnabarinus* (Boisduval)^21^, larvae of *Tuta absoluta* (Meyrick)^22^ and antifeedant effects against the aphid *Myzus persicae* (Sulzer)^23^. Moreover, many compounds were identified in different *D. viscosa* extracts, but very few of them have been associated with specific biological activity. However, until now, to the best of our knowledge, no studies have been carried out on the effects of *D. viscosa* against stored-product pests.

The granary weevil, *Sitophilus granarius* (L.), is one of the most damaging pests of stored grains that causes both quantitative and qualitative losses due to insect feeding on grains, alteration of nutritional and aesthetic value and contamination of commodities with insect bodies, excrement and mycotoxins that result from insect-promoted fungal growth during storage^24–29^. Control of the granary weevil is difficult due to the endophytic development of immature stages that are well protected within grains from pesticides, the increasing legislation limits to the use of some fumigants and broad-spectrum contact insecticides, and the increasing consumer demand for safe food^30^.

In this study, firstly the bioactivity of *D. viscosa* to *S. granarius* adults was evaluated by ingestion and contact toxicity assays with different plant extracts; later, by partially purifying the most active extract (n-hexane), the compounds present in the fraction retaining toxicity were identified. In parallel, both *D. viscosa* extracts and active subfractions were also evaluated for their effect on AChE in order to have a preliminary indication about their mechanism of action.

## Results

### Bioactivity of plant crude extracts

To gain a first insight into biological activity of *D. viscosa* against *S. granarius* adults, in a first series of experiments the ingestion toxicity and nutritional effects of plant extracts were evaluated by a flour disk bioassay^31^. Thus, *S. granarius* adults were fed for 5 days with flour disks treated with n-hexane, methanol or water extracts of plant aerial part, or the solvent alone (control). At the end of this period, mortality rate (%), insect relative growth rate (RGR), relative consumption rate (RCR), efficiency conversion of ingested food (ECI) and feeding deterrent index (FDI) were calculated (Tables 1–3). In the dose range tested, almost no ingestion toxicity was observed for all the extracts. Moreover, no significant differences in nutritional parameters were found in the treatment with n-hexane and methanol extracts. Only the highest dose of the aqueous extract caused a significant reduction of RGR and RCR indexes compared with control values, however the resulting ECI was not found to be significantly different from control. FDI was only slightly affected by different doses of the aqueous extract.

**Table 1:** Mortality (Mort.), feeding deterrent index (FDI), relative growth rate (RGR), relative consumption rate (RCR) and efficiency conversion of ingested food (ECI) of *S. granarius* adults fed for 5 days on flour disks treated with increasing concentrations of *D. viscosa* n-hexane extract. No significant differences at P = 0.05 (Tukey HSD test) were found.

Concentration (µg/disk)	Mort. (%)	FDI (%) ± S.E.	RGR (mg/mg/day) ± S.E.	RCR (mg/mg/day) ± S.E.	ECI (%) ± S.E.
750.00	8	−12.939 ± 6.924	0.020 ± 0.002	0.311 ± 0.007	6.543 ± 0.631
375.00	6	−11.066 ± 7.622	0.025 ± 0.003	0.310 ± 0.012	8.042 ± 0.670
187.5	2	−13.591 ± 6.605	0.020 ± 0.003	0.308 ± 0.011	6.302 ± 0.726
93.75	0	−1.318 ± 10.068	0.021 ± 0.002	0.285 ± 0.032	7.578 ± 0.950
46.87	2	−11.295 ± 5.552	0.042 ± 0.002	0.352 ± 0.044	10.295 ± 3.122
Control	0	—	0.015 ± 0.002	0.294 ± 0.012	4.913 ± 0.838
F		0.727	1.476	0.676	1.222
*d.f*.		4	5	5	5
P		0.584	0.234	0.646	0.32

**Table 2:** Mortality (Mort.), feeding deterrent index (FDI), relative growth rate (RGR), relative consumption rate (RCR) and efficiency conversion of ingested food (ECI) of *S. granarius* adults fed for 5 days on flour disks treated with increasing concentrations of *D. viscosa* methanol extract. No significant differences at P = 0.05 (Tukey HSD test) were found.

Concentration (µg/disk)	Mort. (%)	FDI (%) ± S.E.	RGR (mg/mg/day) ± S.E.	RCR (mg/mg/day) ± S.E.	ECI (%) ± S.E.
750.00	0	0.248 ± 4.612	0.026 ± 0.003	0.217 ± 0.006	12.008 ± 1.762
375.00	0	−7.653 ± 4.668	0.024 ± 0.001	0.223 ± 0.007	10.747 ± 0.509
187.5	0	−10.770 ± 4.946	0.020 ± 0.001	0.243 ± 0.008	8.433 ± 0.377
93.75	0	−11.280 ± 4.096	0.023 ± 0.001	0.235 ± 0.009	9.901 ± 0.728
46.87	0	−10.720 ± 3.893	0.025 ± 0.003	0.247 ± 0.011	10.007 ± 0.961
Control	0	—	0.023 ± 0.015	0.216 ± 0.017	8.742 ± 6.270
F		0.180	0.085	1.650	0.234
*d.f*.		4	5	5	5
P		0.350	0.994	0.185	0.944

**Table 3:** Mortality (Mort.), feeding deterrent index (FDI), relative growth rate (RGR), relative consumption rate (RCR) and efficiency conversion of ingested food (ECI) of *S. granarius* adults fed for 5 days on flour disks treated with increasing concentrations of *D. viscosa* water extract. Values in the same column followed by the same letters are not significantly different at P = 0.05 (Tukey HSD test).

Concentration (µg/disk)	Mort. (%)	FDI (%) ± S.E.	RGR (mg/mg/day) ± S.E.	RCR (mg/mg/day) ± S.E.	ECI (%) ± S.E.
750.00	0	11.553 ± 2.871 b	0.009 ± 0.002 a	0.248 ± 0.008 a	3.741 ± 0.722 a
375.00	0	6.832 ± 3.962 ab	0.011 ± 0.004 ab	0.255 ± 0.011 ab	4.195 ± 1.732 ab
187.5	0	−9.497 ± 6.794 a	0.021 ± 0.001 ab	0.296 ± 0.006 bc	7.000 ± 0.236 ab
93.75	0	1.635 ± 3.109 ab	0.023 ± 0.002 b	0.279 ± 0.008 abc	8.165 ± 0.237 b
46.87	0	7.019 ± 4.898 ab	0.021 ± 0.003 ab	0.279 ± 0.007 abc	7.473 ± 0.881 ab
Control	0	—	0.024 ± 0.004 b	0.297 ± 0.015 c	7.882 ± 1.098 ab
F		3.142	4.372	4.591	3.586
*d.f*.		4	5	5	5
P		0.037	0.006	0.004	0.010

Having found poor or no effect of *D. viscosa* extracts by ingestion, in another series of experiments the contact toxicity of same extracts was evaluated by topic application^32,33^. Differently from water extract, which caused no mortality 24 h after treatment, a significant contact toxicity was recorded for both n-hexane and methanol extracts. However, a difference between the two active extracts was found, being the former much more active (80% mortality at the highest dose) than the latter (only 25% at the same dose). The LD_50_ value was 53.20 μg/adult for the n-hexane extract (Table 4).

**Table 4:** Insect mortality, regression equation and LD_50_ value of *D. viscosa* aerial part extracts 24 h after topical application.

*D. viscosa* extract (μg/adult)	Mortality rate (%)^1^ (mean ± SE)	Regression equation	X²	LD_50_ (μg/adult)
n-hexane				
75.0	80.0 ± 11.6 a	y = 4.62 × −7.97	2.27	53.20
37.5	15.0 ± 9.6 b			
18.8	5.0 ± 5.0 b			
9.4	0.0 ± 0.0 b			
4.7	0.0 ± 0.0 b			
0.0	0.0 ± 0.0 b			
F	*23.920*			
*d.f*.	5			
P	<*0.01*			
methanol				
75.0	25.0 ± 12.6 a	NA	NA	NA
37.5	10.0 ± 5.8 ab			
18.8	5 ± 5.0 b			
9.4	0.0 ± 0.0 b			
4.7	0.0 ± 0.0 b			
0.0	0.0 ± 0.0 b			
F	*2.677*			
*d.f*.	*5*			
P	*0.06*			
water				
0.0–75.0	0 ± 0.0	NA	NA	NA

^1^Data were submitted to ANOVA followed by Tukey HSD test. For each extract, values followed by the same letters are not significantly different at P = 0.05. The mean adult weight was 1.983 ± 0.018 mg. NA, not applicable.

### Partial purification of n-hexane extract and bioactivity of its subfractions

**Figure 1:**
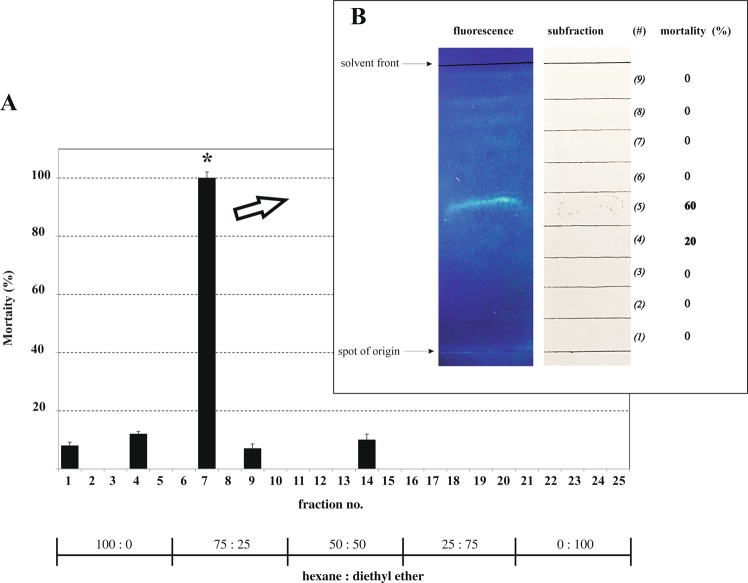
(**A**) Mortality (%) of *S. granarius* adults 24 h after topic application of different fractions of *D. viscosa* n-hexane extract (0.5 μL/adult) obtained by silica gel column chromatography (*P < 0.05, n = 3). (**B**) Thin layer chromatography of fraction 7 and insect mortality (%) caused by the different TLC subfractions: aliquot (100 μL) of the active fraction (no. 7) showed in A was fractioned on a silica gel plate (n-hexane:diethyl ether, 80:20, as eluent) and observed by UV lamp (254–365 nm); recovered subfractions (2 mL) were concentrated (100 μL) and used for contact toxicity bioassay as in A.

In the light of its highest contact toxicity, n-hexane extract was deeper analyzed in order to identify the active compounds contained in this mixture. Thus, an aliquot of this extract was purified by silica gel column chromatography and 25 fractions recovered and checked for contact toxicity against granary weevils. Toxicity was almost completely restricted to fraction 7 which returned a 100% mortality value (Fig. 1A). Therefore, this fraction 7 was further purified by means of thin layer chromatography and the recovered TLC subfractions tested on the insect as above (Fig. 1B). The highest insect mortality (60%) was obtained with the subfraction #5 which showed a clear fluorescent spot (Rf = 0.55). A lower toxicity was also found for subfraction #4, while no toxicity was observed for the other subfractions.

### Chemical analyses of active fractions

**Figure 2:**
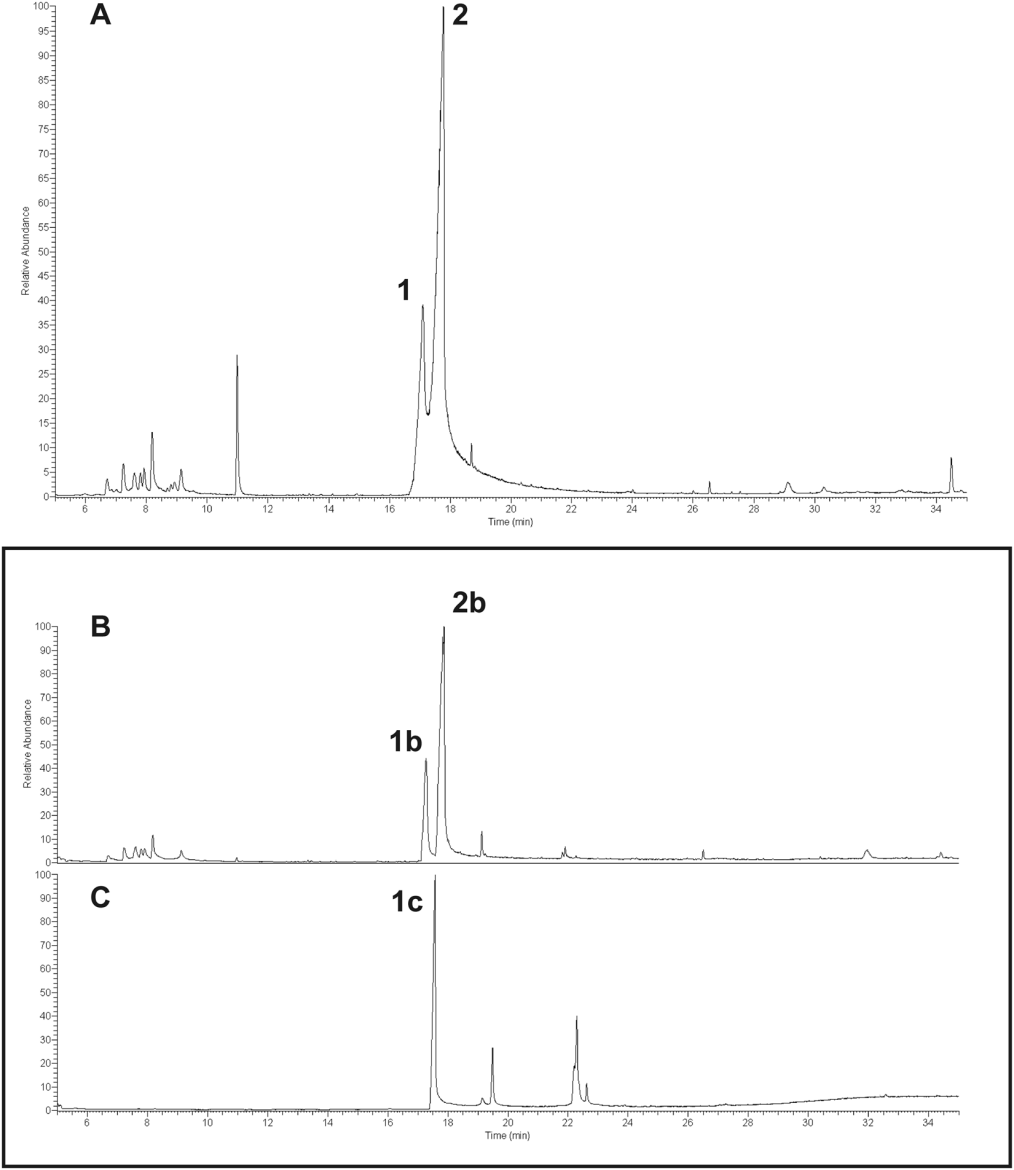
Total ion current chromatograms from GC-MS analysis of real samples: (**A**) fraction 7 of *D. viscosa* n-hexane extract obtained by silica gel column chromatography; (**B**) subfraction #5 obtained by TLC of fraction 7 showed in A; (**C**) a standard of β-costic (2 ng µL^−1^). Peak 1 and 1b = α-costic acid, Peak 2 and 2b = γ-costic acid, Peak 1c = β-costic acid.

**Figure 3:**
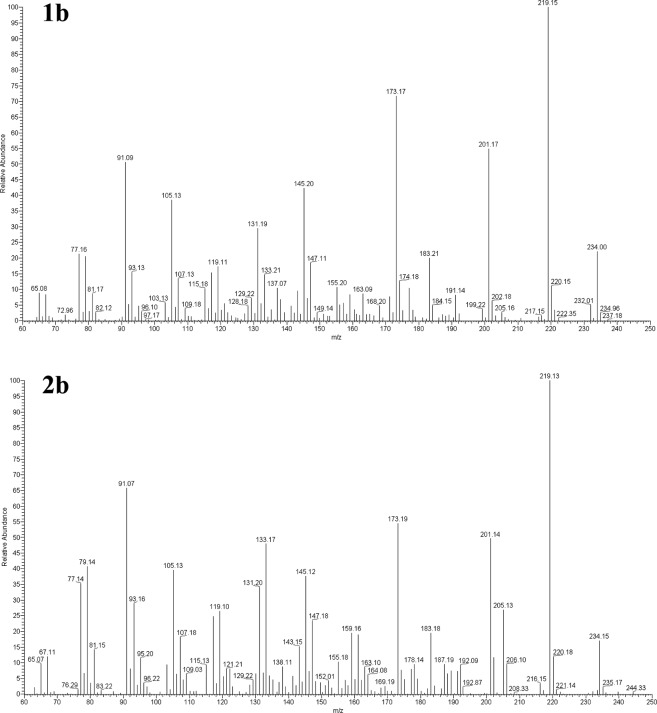
Mass spectra relative to peak 1b (α-costic acid), peak 2b (γ-costic acid) of Fig. 2. Scan acquisition in positive chemical ionization was from m/z 60 up to 400 a.m.u. at 1.68 scan s^−1^ and 70 eV.

**Figure 4:**
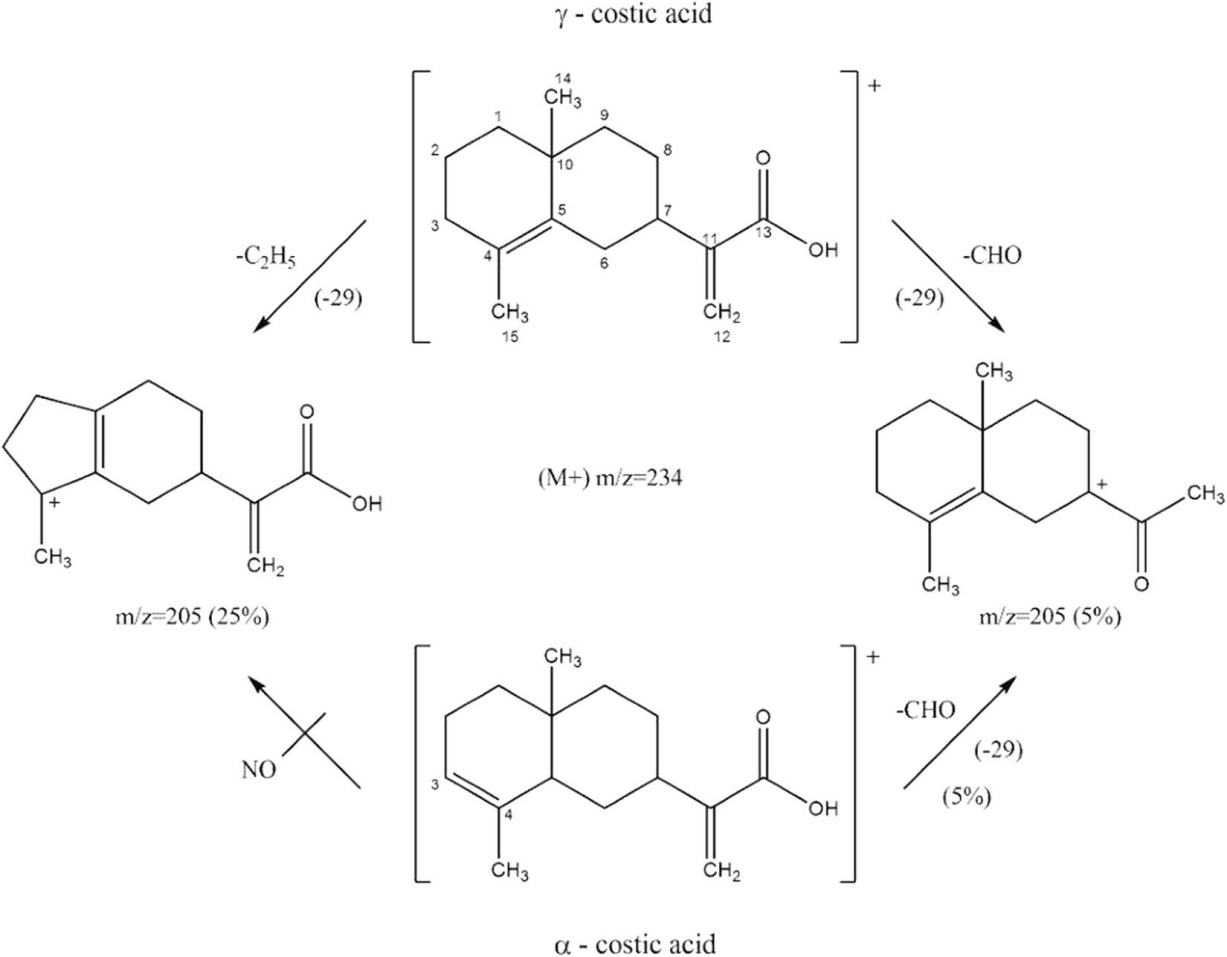
Characteristic fragmentation of α- and γ-costic acid isomers.

Having the biological assays identified the fraction and subfractions retaining n-hexane extract toxicity, a chemical characterization of these ones was carried out. The GC-MS analysis of fraction 7 revealed the presence of two main peaks (24.7 and 67.1% relative abundance, respectively) which were putatively identified as costic acid (Fig. 2A). Moreover, these peaks were also found in both bioactive subfractions (# 4 and 5), with more abundance in the most active one (Fig. 2B). To further ascertain the exact molecular identification of compounds revealed in Fig. 2A, a comparison with a standard of β-costic acid was carried out (Fig. 2C). Observing the mass spectra relative to peaks 1b and 2b (Fig. 3), there is a noticeable difference in mass fragment 205 (m/z). In fact, in spectrum 2b, this fragment (30%) is greater than that in spectrum 1b (5%). These differences are related to the structures of the two costic acid isomers. The mass fragment 205 observed in spectrum 2b comes from the molecular peak M^+^ (234) after the loss of a CHO (29) fragment or that of a C_2_H_5_ (29) fragment; the mass fragment 205 in spectrum 1b comes from the molecular peak M^+^ (234) after the loss of only the CHO molecule (29), whereas the other loss is prevented by the presence of double bond, in position (3–4) which prevents the formation of a pentane ring. Thus, the α-costic acid is assigned to peak 1b and the γ-costic acid isomer to peak 2b, respectively. The fragmentation scheme is shown in Fig. 4. Further, under these experimental conditions, and with this stationary phase, the order of elution of the costic acid isomers from the capillary column is α- (RT = 17.28 min), β- (RT = 17.57 min) and γ-costic acid (RT = 17.86 min), thus confirming what reported by other authors (in particular Fig. 8 of the cited paper)^34^.

Finally, by comparison with β-costic acid standard, the amount of α- and γ-costic acid was found to be respectively 6.802 and 19.144 mg in the fraction 7, 0.471 and 1.283 mg in TLC subfraction #5, 0.188 and 0.513 mg in TLC subfraction #4. Accordingly, in contact toxicity assay (Fig. 1) the calculated dose of α- and γ-costic was respectively 3.40 and 9.57 μg/adult for fraction 7 (mortality 100%), 2.36 and 6.42 μg/adult for TLC subfraction #5 (mortality 60%), 0.94 and 2.57 μg/adult for TLC subfraction #4 (mortality 20%).

### AChE assay

**Figure 5:**
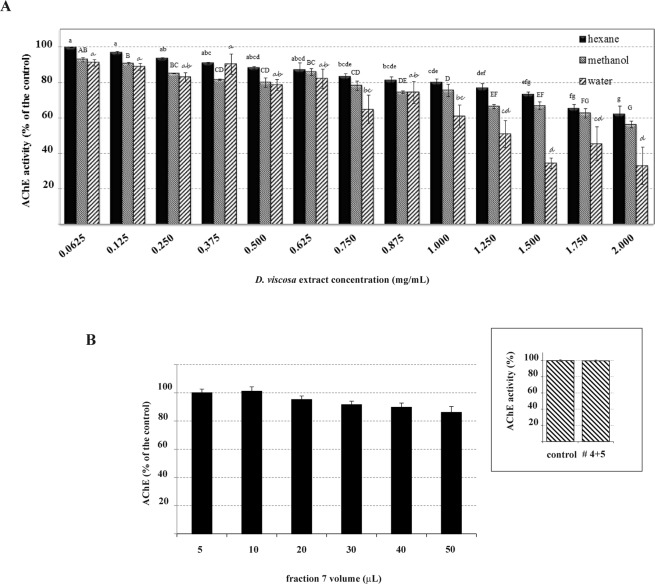
(**A**) Mean values (±SE) of AChE activity obtained in the presence of different concentrations of n-hexane, methanol and water extracts of *D. viscosa*. Values (3 for each concentration) were calculated as % of the control (enzyme activity measured in the absence of plant extract). (B) Mean values (±SE) of *S. granarius* AChE obtained in the presence of either different volume of fraction 7 (whose content in costic acid was 25.9 µg/µL) or subfractions #4 + 5 (50 μL, about 0.6 mg costic acid) (inset). Different letters within the same font indicate a significant difference (P < 0.05).

Being the impairment of nervous system function the main mechanism by which plant metabolites toxicity occurs^35,36^, the effect of plant extracts, fractions, and subfractions on the AChE, the most conserved mechanism in nervous transmission, was investigated. A progressive inhibition of pure commercial AChE was found at increasing concentration of each extract (Fig. 5). At maximum concentration checked (2 mg/mL) the water extract showed the highest capability (about 70% inhibition), whereas n-hexane extract was the less effective one (about 40% inhibition) (Fig. 5A). Differently, from plant n-hexane extract, no inhibition of this enzyme was registered for any silica gel column fractions (Supplementary Fig. S2). Moreover, to confirm the low anticholinesterase activity of n-hexane fraction, the effect of both fraction 7 and its active subfractions was further checked on AChE activity obtained from adult granary weevils (Fig. 5B). A very poor inhibition was found for increasing volumes of fraction 7, corresponding to 0.129–1.293 mg/ml range of costic acid (both isomers), whereas insect enzyme was found to be insensitive to the purified TLC active subfractions (Fig. 5B inset).

## Discussion

Results of this study confirm *D. viscosa* as an important source of biologically active compounds^11,12^ and, more importantly, suggest the possible application of this weed plant against stored-product pests. This consideration mostly relies on the high contact toxicity exerted by the n-hexane extract of *D. viscosa* aerial part against *S. granarius* adults. In fact, contact toxicity was low or absent respectively for methanol and water extracts and no ingestion toxicity was found for any of the three extracts. Marked differences in the toxicity of plant extracts obtained using organic solvents of different polarity was also observed in many previous studies^9,32^ including plant extracts of species belonging to the Asteraceae family^10^.

Nutritional parameters of insects fed with untreated flour disks (control) were in fairly good agreement with those reported in other studies^37^. Plant extracts exerted negligible effects on nutritional parameters of adult weevils except for the highest dose of water extract that caused a significant reduction of food intake (RCR) and relative growth rate (RGR) when compared to control. Since no ingestion toxicity was observed this alteration in nutritional parameters indicates the presence of antifeedant constituents in the water extract (rather than post-ingestion toxicants) which more likely act on the gustatory organs of the mouthparts^38^. However, chemical analysis of water extract is needed to support or refute this hypothesis, as well as for better understanding the variability registered with this extract for the other nutritional indexes. Moreover, the knowledge of both methanol and water extracts composition would shed some light on the different effects exerted by the three plant extracts.

On the other hand, the main outcome of this study remains the high contact toxicity of n-hexane extract. This toxic activity was comparable with those observed for n-hexane extract of *Scrophularia canina* L. (LD_50_ 75.22 μg/adult)^39^ against the same insect species, and similar to those reported for extracts of other plants proposed to have potential toxic activity against the congener *S. zeamais* Motschulsky^40,41^.

GC-MS analysis of active fractions strongly suggested costic acid, and in particular α- and γ- isomers, as the compound responsible for the contact toxicity of n-hexane extract against the granary weevil adults. Accordingly, an increasing insect mortality was observed in response to fractions containing increasing amounts of α- and γ- costic acid isomers. In this regard, the amount of costic acid (sum of both isomers) in fraction 7 was calculated to be 25.9 mg. Since this fraction comes from 187.5 mg of n-hexane extract loaded on silica gel column chromatography, it can be assumed that the amount of costic acid in this extract was at least 13.8%. Such a value is in fairly good agreement with the 10.6% found in a similar extract of the same plant by other authors^42^.

Identification of costic acid isomers in *D. viscosa* appears to be a controversial matter. In this study, the n-hexane extract was proved to contain α- and γ-costic acid, but not the β- isomer. On the contrary, this latter compound was identified and showed to be active against the mite *Varroa destructor* (Anderson and Trueman)^43^. However, in other studies some authors identified the α-, but not β-isomer^44,45^, whereas others identified both α- and β- isomers^20,42^. Whether these differences rely on different geographical region of plant growth, more unlikely on the different extraction conditions, is a matter of speculation.

In this study, topical application of a standard of β-costic acid in the dose range of 0.5–50 μg/adult did not cause insect mortality (data not reported), thus suggesting a strict dependence of the observed toxicity on the costic acid isomerism. Notice that this high specifity would not be unique since the same applies for alantolactone isomers of *Inula helenium* (L.) which showed a sharp decrease in toxic activity on both larvae and adults of *Aedes aegypti* due to the shift of double bond outside the carbon ring, as occurs for β-costic acid^46^. Future studies with α- and γ-costic acid standards, not commercially available at present, should clarify the relative contribution of each isomer to the contact toxicity of *D. viscosa* n-hexane extract.

Plant extracts investigated here showed a moderate dose dependent AChE inhibitory capability, in fair agreement with previous studies in which anticholinesterase activity for *D. viscosa* extracts and essential oil was reported^47,48^. However, the active fraction, and subfractions, of n-hexane extract induced a very poor inhibition of both insect and pure AChE, thus ruling out this mechanism of action. This lack of anticholinesterase activity appears of particular interest in the light of the continuous search for new compounds acting on target/s different from AChE^49^. It is known, in fact, that nervous transmission impairment, especially through AChE inhibition, have represented historically the main target of insecticides^35^. On the other hand, the occurrence of cholinergic system in almost all animals^50^, as well as the development of pesticide resistance and tolerance in insects mostly due to AChE mutation and/or duplication^51^, recently led to a sharp restriction of the use of AChE inhibitors^52^. Hence, results reported here suggest *D. viscosa* n-hexane extract as a valid natural and ecofriendly tool for the control of stored-product pests.

## Materials and Methods

### Insect

*S. granarius* was reared for several generations on wheat grains stored in glass cylindrical containers (Ø 15 × 15 cm) closed by metallic screen (1 mm mesh) and maintained in the dark in a climatic chamber set at 22±2 °C and 60±5% R.H. Adults two-four weeks old of mixed sexes were used for the experiments. For antifeedant and nutritional bioassays, insects were starved for 24 h before use.

### Plant Material

About 300 plants of *D. viscosa* (100 plants/sampling) were collected during the flowering stage (October/November) from rural areas near Campobasso (Molise region, Italy) at 650 m a.s.l. (Supplementary Fig. S1). A voucher specimen was deposited in the herbarium of Department of Agricultural, Environmental and Food Sciences, University of Molise. For each plant, the aerial part containing at least 15 flowers and 10 leaves were dried and ground to a fine powder. Aliquots (50 g) of the dry powder were extracted with different solvents (600 mL) of increasing polarity, n-hexane, methanol, water (99% purity, Sigma-Aldrich Milan, Italy), for 24 h at room temperature. Each crude extract was centrifuged (30,000 × g, 15 min), and the supernatant paper filtered (Whatman No. 113). N-hexane and methanol extracts were dried under vacuum in a rotary evaporator (Laborota 4000, Heidolph, Germany) whereas water extract was lyophilized (Hetosicc FD 2.5, De Mori, Milan, Italy) until used. The residues obtained were 39.0, 129.7, and 192.3 g/kg dry weight for n-hexane, methanol and water extracts, respectively. Residues were stored at –20 °C until used.

### Antifeedant and Nutritional Activity

Effects of *D. viscosa* extracts on the feeding activity and nutrition of granary weevil adults were evaluated by the flour disk bioassay^31^. Wheat flour (10 g) was uniformly suspended in distilled water (50 mL) by stirring. To obtain flour disks, aliquots (200 μL) of suspension were dropped onto a plastic Petri dish and left overnight at 26 ± 2 °C and 60 ± 5% R.H. to dry. In parallel, plant extracts samples were prepared by dissolving the residues of n-hexane, methanol and water extracts in n-hexane, acetone:methanol (1:1) and acetone:water (1:1), respectively. For each sample two-fold serial dilutions were prepared. Thus, disks were treated with sample solutions (5 μL) corresponding to different concentrations (750.00, 375.00, 187.50, 93.75, 46.87 μg/disk) or the solvent alone as control. Disks were held at room temperature for 2 h for solvent evaporation. In a pre-weighed glass vial (Ø 2.5 × 4.0 cm) 2 flour disks were introduced and the weight measured; later 10 group-weighed weevil adults were added and each vial was then re-weighed and maintained in the dark at 26 ± 2 °C, 60 ± 5% R.H. for 5 days. At the end of the test, for each glass vial, insects were removed, the number of dead insects recorded, and the weight of both the 2 flour disks residues and live insects were separately measured. As a control, glass vials containing treated flour disks but without insects were prepared to determine any decrease in weights due to evaporation of solvent and sample. For each sample concentration, as well as for control, 5 replicates were set up. The following nutritional indices^40,53^ for each replicate were calculated:

Relative Growth Rate (RGR) = (A − B)/B × day^−1^

Relative Consumption Rate (RCR) = D/B × day^−1^

Efficiency Conversion of Ingested food (ECI) = (RGR/RCR) × 100

Feeding Deterrence Index (FDI) (%) = [(C − T)/C] × 100

where A = mean weight (mg) of live insects on fifth day;

B = original mean weight (mg) of insects;

C = consumption of control disks;

D = biomass ingested (mg)/no. of living insects on the fifth day;

T = consumption of treated disks.

Data were submitted to ANOVA followed by Tukey’s HSD test for mean comparisons. Statistical analyses were performed with SPSS (Statistical Package for the Social Sciences) v.23 for Windows (SPSS Inc., Chicago, IL).

### Contact toxicity

The contact toxicity of *D. viscosa* extracts to granary weevil adults was determined by topical application as reported in^32,33,39^ with minor differences. Residues of the n-hexane extract, its (sub)fractions, and β-costic acid (standard, Chem Faces, China) were dissolved in n-hexane, whereas methanol and water extracts were dissolved in acetone:methanol (1:1) and acetone:water (1:1), respectively. For each sample, two-fold serial dilutions for plant extracts (150.00–9.36 μg/μL) and β-costic acid standard (100.00–1.00 μg/μL) were prepared. A 0.5 μL droplet of each solution was applied onto the pronotum of an adult weevil in thanatosis using a Hamilton’s syringe (700 series, MicroliterTM Hamilton Company, USA). For each sample, 60 insects divided in 12 replicates were used. Concentrations were expressed as μg of sample per adult (average adult weight 1.98 ± 0.02 mg). Insects treated with solvent alone (n-hexane, acetone/methanol and acetone/water) were used as control. After topical application, the insects were confined in a Petri dish within a metal ring (Ø 4.0 × 2.5 cm) covered with metallic net (1 mm mesh) to prevent insects escape, provided with 5 wheat kernels and maintained in the dark at 26 ± 2 °C and 60 ± 5% R.H. The number of dead insects was recorded after 24 h. The percentage mortalities were transformed to arcsine square-root values for one-way analysis of variance (ANOVA). Treatment means were compared and separated by Tukey HSD test. The median lethal dose (LD_50_) values, the confidence upper and lower limits, regression equations and chi-square (χ^2^) values were calculated using probit analysis^54^.

### Silica Gel Column Chromatography

Aliquots (250 mg) of the oily residue of the n-hexane extract were dissolved in n-hexane (1 mL) and 750 μL of this mixture (about 187.5 mg of n-hexane extract residue) were loaded on a glass chromatographic column (Ø 1.5 cm, 20 cm height) packed with n-hexane slurry of silica gel S previously activated (120 °C for 1 h). The column was eluted with increasing concentrations of diethyl ether in n-hexane and 25 fractions (20 mL each) were collected. Each fraction was first concentrated to 1 mL under a gentle flow of nitrogen, then, to remove diethyl ether, fractions were added with n-hexane (1.5 mL) and again concentrated (as above) to a final concentration of 1 mL. These solutions were tested for their contact toxicity against *S. granarius* adults and anti-AChE activity (see below). An aliquot (10 μL) of the active fraction was diluted 1:500 and analyzed in GC-MS.

### Thin Layer Chromatography (TLC)

An aliquot (100 μL) of the most active fraction against insects was further fractioned on a silica gel plate (5 × 20 cm, thickness 0.25 cm, Merck) by means of an ascending chromatography using a mixture of diethyl ether (20%) in n-hexane as eluent. The plate was observed by UV lamp (UVSL-15, Multi-Band, California, USA) at long and short wave-UV (254, 365 nm, respectively) and divided into 9 subfractions which were recovered with 10 mL of n-hexane:diethyl ether (80:20). Each subfraction was concentrated to 2 mL and an aliquot (50 μL) diluted 1:50 was used for GC-MS analysis. To assess insecticidal activity, subfractions (1.95 mL) were further concentrated under a gentle flow of nitrogen to 100 μL.

### GC/IT-MS Analysis and Quantification

A gas chromatograph Finnigan Trace GC Ultra equipped with an ion-trap (IT) mass spectrometry (MS) detector Polaris Q (Thermo Fisher Scientific, Waltham, MA), a Programmed Temperature Vaporizer (PTV) injector and a PC with a chromatography station Xcalibur (Thermo Fisher Scientific), was used. A fused-silica capillary column with chemically bonded phase (SE-54, 5% phenyl–95% dimethylpolysiloxane) was prepared in our laboratory^55–57^ with the following characteristics: 30 m × 0.25 mm i.d., *N* (theoretical plate number) 115,000 for *n*-dodecane at 90 °C; *K*’ (capacity factor) 6.7; d_f_ (film thickness) 0.23 μm; u_opt_ (optimum linear velocity of carrier gas, hydrogen) 38.5 cm s^−1^, and *UTE*% (utilization of theoretical efficiency) 89%.

Helium was used both as carrier gas at a flow rate of 1 mL min^−1^ and dumping gas in the ion-trap at 0.3 mL min^−1^. The column oven temperature was programmed from 100 °C to 280 °C (5 min) at 6 °C min^−1^. Injected sample volume was of 1 μL. The PTV injector was performed in splitless mode: it was programmed from 110 °C to 290 °C at 800 °C min^−1^ and cooled after 4 min. The splitter valve was closed for 180 s. The transfer line and ion source were held at 270 °C and 250 °C, respectively. Scan acquisition in positive chemical ionization was from m/z 60 up to 400 a.m.u. at 1.68 scan s^−1^ and 70 eV. The costic acid isomer concentrations were obtained by calibration graphs of area (β-costic acid) plotted versus β-costic acid standard concentration (0.05, 0.1, 1.0, and 5.0 µg mL^−1^). The sample was quantified in triplicate.

### AChE Assay

AChE activity was detected photometrically (λ = 412 nm, 25 °C) by means of a Jasco V-570 spectrophotometer (Tokyo, Japan) according to^58^ by using 5,5’dithio bis(2-nitrobenzoic) acid (DTNB). Briefly, about 0.01 EU from either commercial (from *Electrophorus electricus*, SIGMA) or insect enzyme, obtained by a crude extract of 100 adults^59,60^, were incubated in phosphate buffer (0.1 M, pH 8.00) plus DTNB (0.2 mM) either in the absence or in the presence of different aliquots of *D. viscosa* extracts and subfractions. For this assay, n-hexane extract and its active fraction were re-suspended in phosphate buffer by means of tween 20 (0.5%); control was made that tween 20 (up to 1%) did not affect AChE activity. Reaction was started by the addition of saturating concentration (2.5 mM) of acetylthiocholine iodide and the rate of absorbance change was obtained as tangent to the initial part of the progress curve. Results were expressed as % of the control (reaction rate measured in the absence of plant extract). Data were submitted to ANOVA followed by Tukey’s HSD test for mean comparisons.

## Supplementary information

supplementary info

## Data Availability

The datasets generated during and/or analyzed during the current study are available from the corresponding authors on reasonable request.
