# Supplementary material for: Biological activity of Dittrichia viscosa (L.) Greuter extracts against adult Sitophilus granarius (L.) (Coleoptera, Curculionidae) and identification of active compounds
Source: Sci Rep. 2019 Apr 23;9:6429. doi: 10.1038/s41598-019-42886-4 (PMC6478880; doi:10.1038/s41598-019-42886-4)
Supplement: Supplementary file 1 — supplementary info [file 41598_2019_42886_MOESM1_ESM.pdf]

Biological activity of *Dittrichia viscosa* (L.) Greuter extracts against adult *Sitophilus granarius* (L.) (Coleoptera, Curculionidae) and identification of active compounds

Giuseppe Rotundo[1*], Gianluca Paventi[2*], Antonia Barberio[1], Antonio De Cristofaro[1], Ivan Notardonato[1], Mario V. Russo[1], Giacinto S. Germinara[3]

[1] *Department of Agricultural, Environmental and Food Sciences - University of Molise, via de Sanctis, 86100 Campobasso, Italy*
[2] *Department of Medicine and Health Sciences "V. Tiberio"- University of Molise, via de Sanctis, 86100 Campobasso, Italy*
[3] *Department of the Sciences of Agriculture, Food and Environment - University of Foggia, Via Napoli 25, 71100 Foggia, Italy*

*corresponding authors:
- Giuseppe Rotundo, rotundo@unimol.it, 0039(0)874 404869
- Gianluca Paventi, paventi@unimol.it, 0039(0)874 404779, http://orcid.org/0000-0003-3673-0091

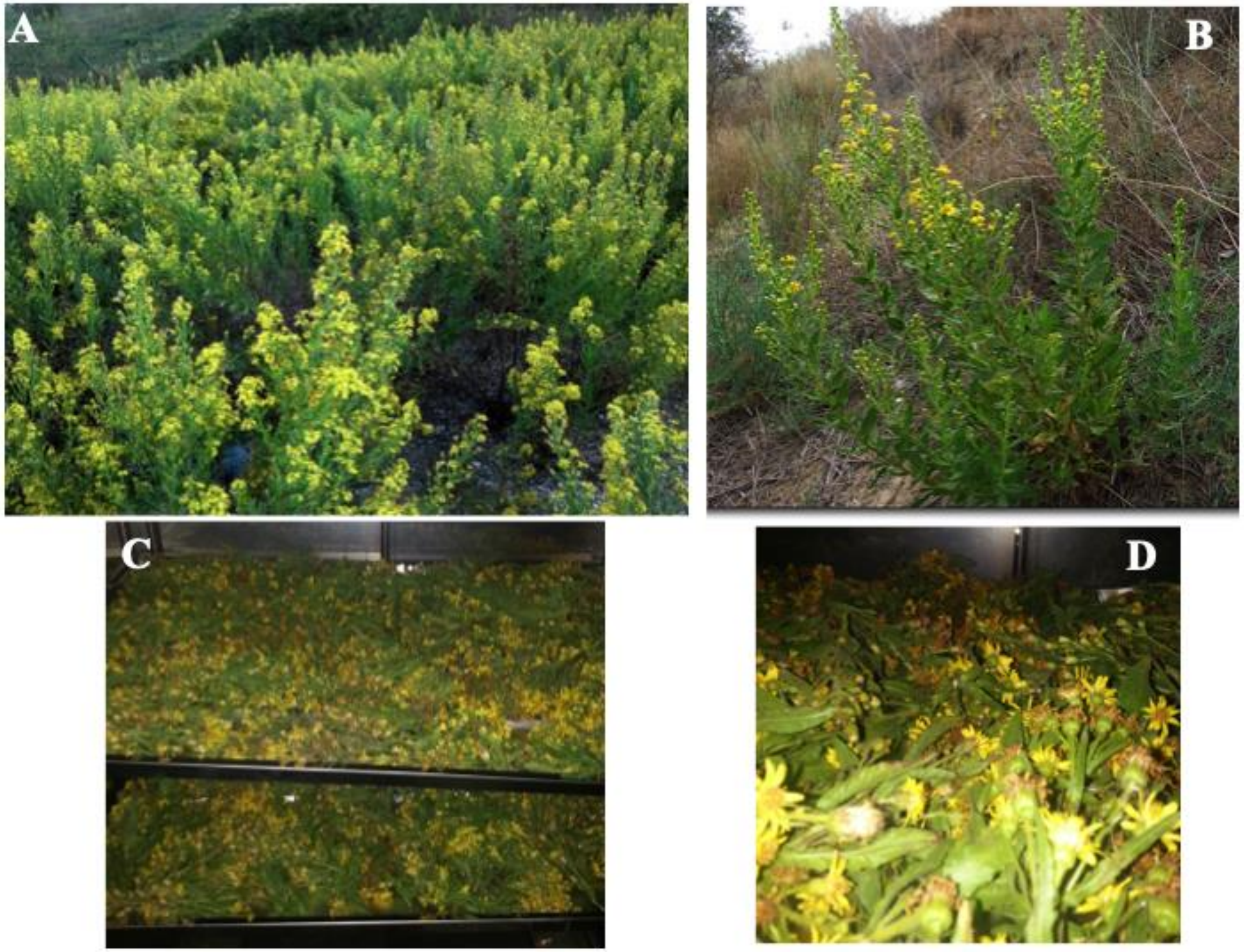

Supplementary Fig. S1. *D. viscosa* plants (insets A, B) in the site of sampling, a rural area near Campobasso, Molise region, Italy (650 m a.s.l.); particular showing the aerial part collected to be dried and ground for solvent extraction (insets C, D).

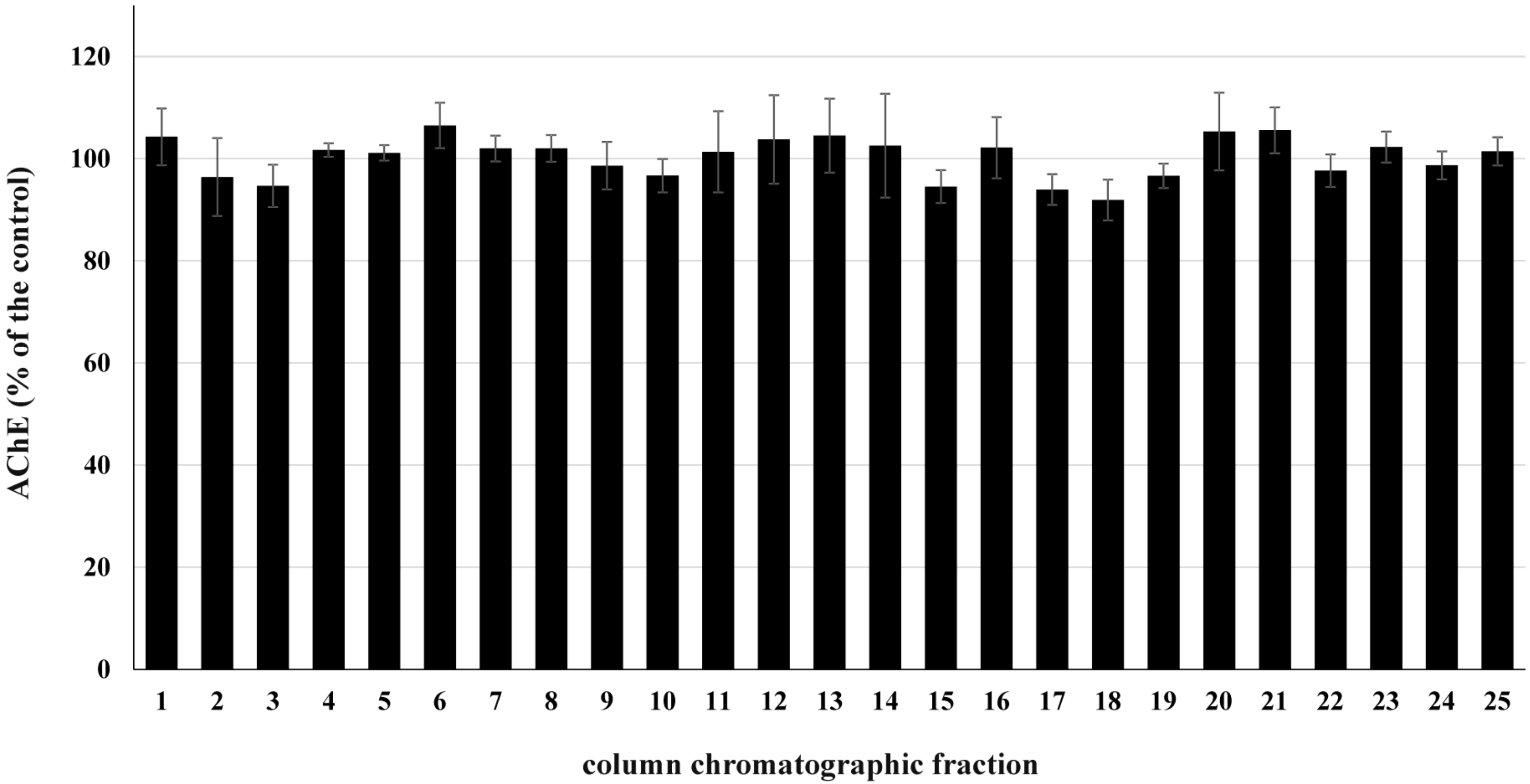

Supplementary Fig. S2. Mean values (± SE) of AChE activity (commercial enzyme) obtained in the presence of different chromatographic fractions (3 mg/ml) of n-hexane extract of *D. viscosa*. Values (3 for each fraction) were calculated as % of the control (enzyme activity measured in the absence of fraction). No significant differences at P = 0.05 (Tukey HSD test) for all fractions were found.
